# The Death of Charlotte Bronte

**Published:** 1857-12

**Authors:** 


### ECLECTIC DEPARTMENT,

### AND SPIRIT OF THE MEDICAL PERIODICAL PRESS

*The Death of Charlotte Bronté.*—Dr. Walter Channing has an article under this title in the Boston Medical and Surgical Journal. Its purpose is to show that it is the duty of the physician, in cases of pregnancy, where the symptoms of that state become morbidly severe, as they did in the case of Charlotte Bronté, endangering the life of the patient, to remove the fœtus. Dr. Channing says:

"It is one of the professional relations of our subject—the treatment of the signs of pregnancy when morbidly aggravated, that we would now speak. Was the cause, the motive cause of those symptoms that produced death in Charlotte Bronté removed? This question is of great interest. Nearly half a century ago, it was our privilege to attend the midwifery lectures of Dr. John Haighton, in London; and a better lecturer than Haighton, is not in our memory. He discussed this question of removing the cause of those symptoms, and showed conclusively that in cases in which other means had failed, and the worst consequences were to be looked for, it was the duty of the physician to remove the cause, viz., to remove the fœtus from the womb. Haighton related his experience, and dwelt on the opposition he had met with in consultations, to such measures as he knew could alone save life. More recently we have spoken with eminent men abroad, on this subject, and have met with objections to this practice; or, when it has been allowed to be proper, it has been after so much evil has been done that there has hardly been any reason to look for success from it. We have felt it our duty to resort to the measure under consideration, and in every case recovery has been rapid and complete."

He goes on to say that he has known death to happen when the measure has been rejected, and when other means have been faithfully used. He names one case in which the mother of the patient refused to consent, unless the physician would guarantee success. This he would not do and the patient died. A clergyman's wife has twice been reduced by nausea and vomiting to excessive weakness, and after full trial of other methods, and it was ascertained that the disease was rapidly increasing, the operation was performed twice, with perfect success. It was also successfully tried in the case of a lady after convulsive movements had occurred in the universal exhaustion. In conclusion, Dr. Channing says:

"We dwell on these cases, because a grave moral question is involved in our subject; and to say that it is only in those cases in which life is clearly in jeopardy, that any physician who deserves the name, would for a moment entertain the question we are considering. It is then as a *remedy*, and only to be used under what we really believe to be desperate circumstances. Whether the cause was removed in Charlotte Bronte's case, or whether she died of pregnancy, we know not. But as the disease continued unrelieved till death, may it not be asked if the cause of that disease did not remain undisturbed till it became the cause of death? The question is put, because in no like case which has come under our care, however unpromising, has death occurred after the removal of the contents of the womb."

